# MicroRNA-939 amplifies *Staphylococcus aureus-*induced matrix metalloproteinase expression in atopic dermatitis

**DOI:** 10.3389/fimmu.2024.1354154

**Published:** 2024-06-05

**Authors:** Jiating Wang, Yejing Huang, Xinfeng Wu, Dongqing Li

**Affiliations:** ^1^ Hospital for Skin Diseases, Institute of Dermatology, Chinese Academy of Medical Sciences & Peking Union Medical College, Nanjing, China; ^2^ Key Laboratory of Basic and Translational Research on Immune-Mediated Skin Diseases, Chinese Academy of Medical Sciences, Nanjing, China; ^3^ Jiangsu Key Laboratory of Molecular Biology for Skin Diseases and STIs, Nanjing, China

**Keywords:** atopic dermatitis, microRNA, *Staphylococcus aureus*, keratinocyte, matrix metalloproteinase

## Abstract

**Background:**

Atopic dermatitis (AD) is a common chronic inflammatory skin diseases that seriously affects life quality of the patients. *Staphylococcus aureus* (*S. aureus*) colonization on the skin plays an important role in the pathogenesis of AD; however, the mechanism of how it modulates skin immunity to exacerbate AD remains unclear. MicroRNAs are short non-coding RNAs that act as post-transcriptional regulators of genes. They are involved in the pathogenesis of various inflammatory skin diseases.

**Methods:**

In this study, we established miRNA expression profiles for keratinocytes stimulated with heat-killed *S. aureus* (HKSA). The expression of miR-939 in atopic dermatitis patients was analyzed by fluorescence in situ hybridization (FISH). miR-939 mimic was transfected to human primary keratinocyte to investigate its impact on the expression of matrix metalloproteinase genes (MMPs) *in vitro*. Subsequently, miR-939, along with Polyplus transfection reagent, was administered to MC903-induced atopic dermatitis skin to assess its function *in vivo*.

**Results:**

MiR-939 was highly upregulated in HKSA-stimulated keratinocytes and AD lesions. *In vitro* studies revealed that miR-939 increased the expression of matrix metalloproteinase genes, including MMP1, MMP3, and MMP9, as well as the cell adhesion molecule ICAM1 in human primary keratinocytes. *In vivo* studies indicated that miR-939 increased the expression of matrix metalloproteinases to promote the colonization of *S. aureus* and exacerbated *S. aureus*-induced AD-like skin inflammation.

**Conclusions:**

Our work reveals miR-939 is an important regulator of skin inflammation in AD that could be used as a potential therapeutic target for AD.

## Introduction

Atopic dermatitis (AD) is a common chronic, recurrent, inflammatory skin disease, usually associated with intense itching and an eczema-like rash. It affects approximately 20% of children and 10% of adults worldwide. The pathogenesis of AD is closely associated with host genetics, skin microbiota disorders, type 2 inflammatory response, and skin barrier disruption (1). Although the type 2 inflammatory response is a major driver in AD, *S. aureus* colonization is the primary reason for infection-induced AD relapse (2, 3). Greater than 90% of AD patients are positive for *S. aureus* colonization in skin lesions. Only 5% of the normal healthy population carries *S. aureus* on their skin, although this percentage may vary based on ethnicity and geographical location (4). Moreover, the density of *S. aureus* in the lesions and non-lesion areas of AD patients is strongly correlated with the severity of the disease (5). Recent studies have indicated that retention of *S. aureus* Agr virulence function during infancy is associated with *S. aureus* skin colonization and the development of AD (6). Various *S. aureus-*derived molecules and byproducts contribute to skin barrier disruption and inflammation, including superantigens, toxins, protein A, proteases, and phenol-soluble modulins (7, 8). In addition, *S. aureus* colonization promotes the development of AD-like skin inflammation in mice (6, 9, 10); however, the precise mechanism for this association with AD remains unclear.

Recent studies of non-coding RNAs have resulted in a greater understanding of the complexity of gene regulation. Of these, miRNAs are approximately 22 nucleotides in length and regulate many cellular processes by acting as post-transcriptional regulators via targeting mRNA degradation and/or translational repression (11). Dysregulation of miRNA expression plays an important role in the pathogenesis of AD. Dicer is a key enzyme in the maturation of miRNAs (12). The absence of Dicer exacerbates skin inflammation and is accompanied by elevated TSLP, which implicates miRNAs in AD pathogenesis (13). MiR-146, which is highly expressed in chronic AD lesions, inhibits the expression of various proinflammatory factors in keratinocytes and ameliorates skin inflammation in AD by targeting the nuclear factor kappa B signaling pathway (14). Furthermore, upregulation of miR-155 expression in patients with AD inhibits CTLA-4 expression and promotes T helper cell proliferation, which in turn, promotes chronic skin inflammation (15). In this study, we aimed to identify the roles of miRNAs in the interactions between *S. aureus* infection and AD.

Recent studies indicate that miR-939 is associated with various malignant tumor types. MiR-939 targets TIMP2 to promote cell proliferation and invasion, and affects the growth of gliomas and non-small cell lung cancer (16, 17). High miR-939 expression predicts a poor prognosis for some cancer patients. LINC00460 can regulate the expression of miR-939 to promote colorectal cancer metastasis (18). In patients with chronic heart failure, miR-939 levels in the serum are increased. TNF-α and iNOS are target genes for miR-939, thus it can regulate inflammatory cytokine-induced apoptosis in endothelial and cardiomyocytes (19); however, the role of miR-939 in AD pathogenesis has not been investigated.

In this study, we demonstrated that miR-939 expression is elevated in HKSA-stimulated keratinocytes and skin lesions of AD patients. MiR-939 increases the expression of matrix metalloproteinases, promotes the colonization of *S. aureus*, and exacerbates *S. aureus*-induced AD-like skin inflammation.

## Results

### MiR-939 is upregulated in heat-killed S. aureus-stimulated keratinocytes and atopic dermatitis

**Figure 1:**
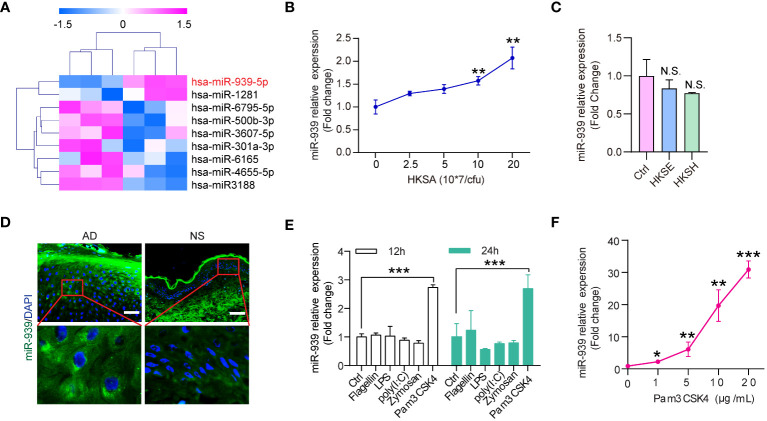
miR-939 is upregulated in heat-killed *S. aureus*-stimulated keratinocytes and atopic dermatitis. **(A)** Differentially expressed miRNA profiles in HKSA-stimulated keratinocytes compared with unstimulated keratinocytes. **(B)** qRT-PCR analysis of the expression of miR-939 in keratinocytes after treated with different concentrations of HKSA. **(C)** qRT-PCR analysis of the expression of miR-939 in keratinocytes after treated with 1x10^8^ CFU of HKSE and HKSH. **(D)** RNA FISH was performed in skin lesions from AD patients (AD) and normal skin from healthy donors (NS). 400x magnification, scale bars: 50 µm. Expression levels of miR-939 after stimulation of keratinocytes with various TLR ligands **(E)** and different doses of Pam3CSK4 **(F)**. The data are presented as mean ± SD. N.S., no significance. *P < 0.05, **P < 0.01, and ***P < 0.001 by One-way ANOVA test.

Skin colonization with *S. aureus* may be a crucial factor in AD pathogenesis. To identify the aberrantly expressed miRNAs in keratinocytes by *S. aureus* colonization, we established miRNA expression profiles for keratinocytes stimulated with heat-killed *S. aureus* (HKSA). The significance analysis of microarrays algorithm was used to analyze the miRNA profiling data. We identified 9 significantly differentially expressed miRNAs (fold change >1.5, p-value <0.05) between HKSA-stimulated keratinocytes and controls (**Figure 1A**). Interestingly, hsa-miR-939–5p was the top upregulated miRNA in HKSA-stimulated keratinocytes (**Figure 1A**). To validate our profiling data, we measured miR-939 expression in keratinocytes stimulated with different doses of HKSA by qRT-PCR. HKSA significantly induced miR-939 expression in keratinocytes in a dose-dependent manner (**Figure 1B**). To study the effects of other Staphylococcal strains on the expression of miR-939, we used heat killed *Staphylococcal epidermidis* (HKSE) and heat killed *Staphylococcal hominis* (HKSH) to treat keratinocytes. We found neither HKSE nor HKSH had any effect on miR-939 expression (**Figure 1C**). To examine the expression pattern of miR-939 in AD, RNA fluorescence *in situ* hybridization (FISH) was carried out with miR-939-specific probes on skin lesion sections obtained from 3 AD patients and 3 healthy individuals. Consistent with the upregulation of miR-939 in HKSA-stimulated keratinocytes, miR-939 expression was significantly increased in the epidermis of the AD samples (**Figure 1D**).

Pathogens secrete pathogen-associated molecular patterns to activate pattern recognition receptors on host cells to activate downstream signaling cascades (20). To determine which receptor in keratinocytes is required for *S. aureus*-induced miR-939 expression, we treated keratinocytes with different TLR ligands, including flagellin (TLR5 ligand), LPS (TLR4 ligand), poly(I: C) (TLR3 ligand), Zymosan (TLR2 ligand), and PamCSK4 (TLR2 ligand) (21, 22) (**Figure 1E**). Pam3CSK4 significantly induced miR-939 expression in a dose-dependent manner (**Figure 1F**). Taken together, these results likely indicate that *S. aureus* activates TLR2 to induce miR-939 expression in AD.

### Transcriptome analysis reveals that miR-939 regulates the HKSA-induced immune response

**Figure 2:**
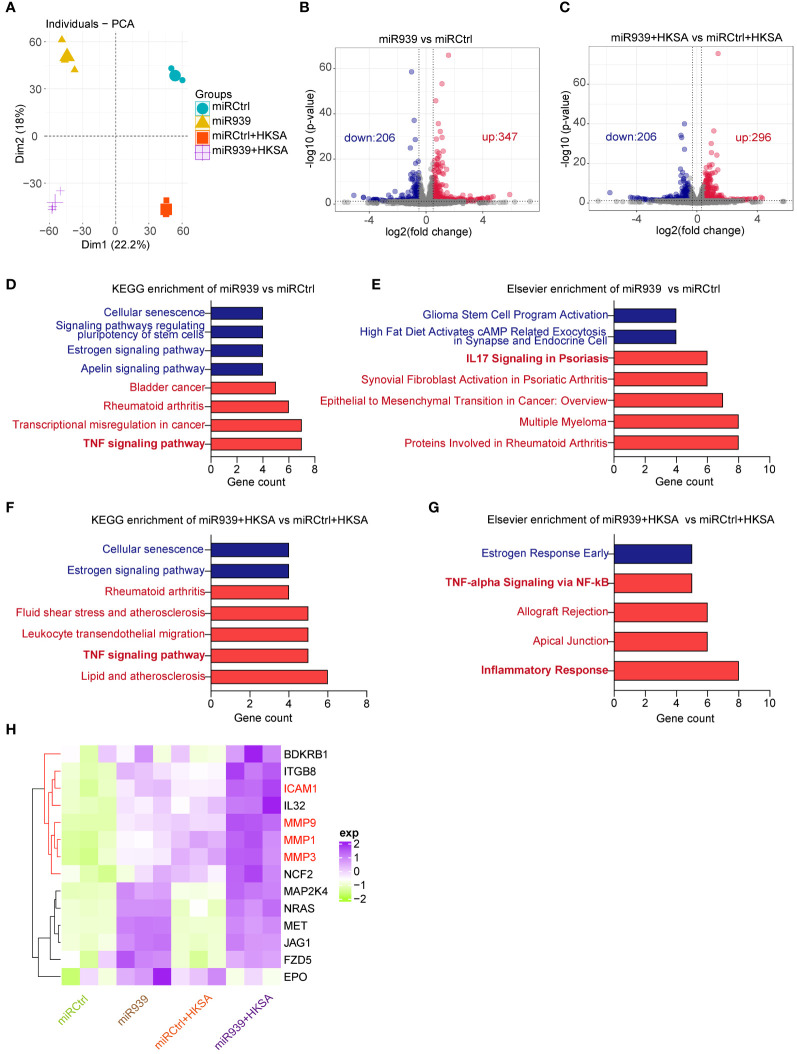
Transcriptome analysis shows that miR-939 regulates HKSA-induced immune response. Keratinocytes transfected with hsa-miR-939–5p mimic or the corresponding negative control, followed by treatment with HKSA or PBS for 8 h were subject to RNA sequencing. **(A)** PCA plot between miRCtrl, miR-939, miRCtrl+HKSA, and miR-939+HKSA. Differentially expressed genes in miR-939 compared with miRCtrl **(B)**, and in miR-939+HKSA compared with miRCtrl+HKSA **(C)** are shown in volcano maps. Top KEGG pathways **(D)** and Elsevier pathways **(E)** for the DEGs regulated by miR-939 as well as the top KEGG pathways **(F)** and Elsevier pathways **(G)** for the DEGs regulated by miR939+HKSA are shown in bar charts. **(H)** Heatmap of 15 top genes upregulated by miR-939.

To study the functional relevance of miR-939 expression under inflammatory conditions induced by HKSA in keratinocytes, we performed a global transcriptomic analysis in keratinocytes with miR-939 overexpression followed by HKSA stimulation. Principal Component Analysis (PCA) indicated that the clustering of samples was categorized into 4 distinct groups designated miRCtrl, miR-939, miRCtrl+HKSA, and miR-939+HKSA, indicating a clear difference in the transcriptome (**Figure 2A**). Subsequently, a two-by-two differential expression analysis of the four groups was performed to evaluate the differentially expressed genes (DEGs) regulated by miR-939 or HKSA in keratinocytes (**Figures 2B, C**; **Supplementary Figures S1A, B**). Kyoto Encyclopedia of Genes and Genomes (KEGG) analysis revealed that cytokine-cytokine receptor interaction, TNF signaling pathway, and IL-17 signaling pathway were enriched in the HKSA-stimulated groups (**Supplementary Figures S1C, D**), which indicates the successful induction of an inflammatory response in keratinocytes by HKSA. Interestingly, these inflammation-related pathways were further enriched in upregulated genes by miR-939 overexpression; however, few enriched pathways were associated with the downregulated genes (**Figures 2D–G**). Therefore, we focused on 15 genes associated with inflammation-related signaling pathways upregulated by miR-939, most of which were upregulated by both miR-939 and HKSA (**Figure 2H**). These results indicate that miR-939 performs a regulatory role in amplifying the HKSA-induced inflammatory response in keratinocytes.

### miR-939 amplifies the S. aureus-induced inflammatory response *in vitro*

**Figure 3:**
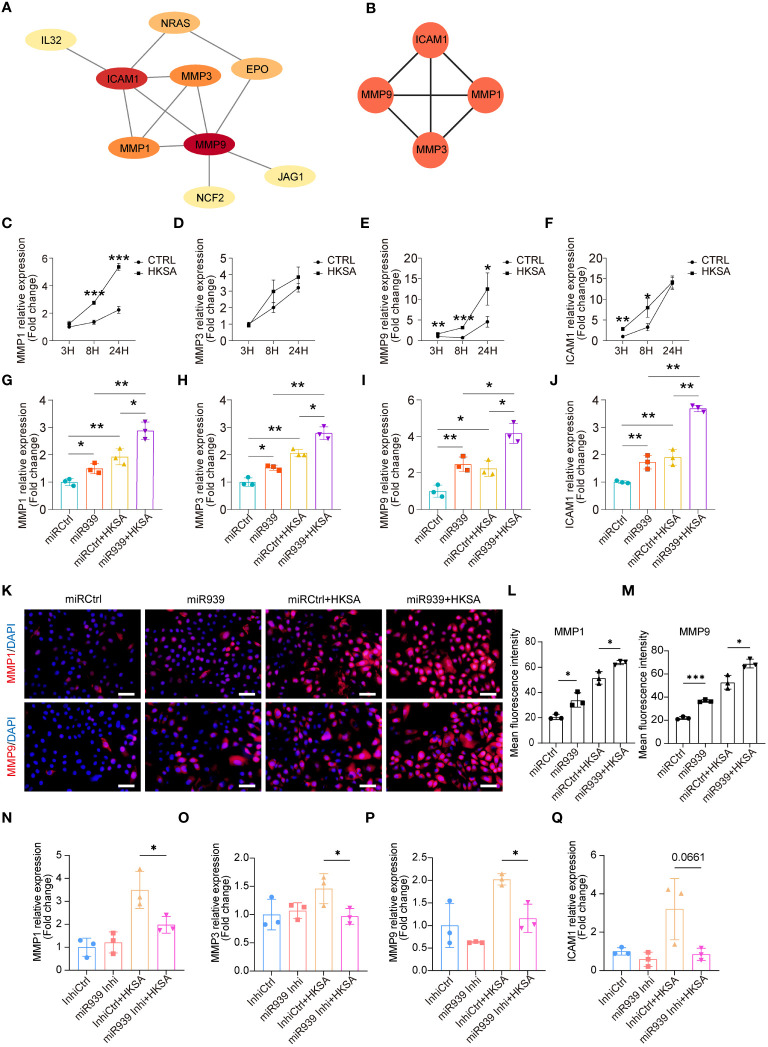
miR-939 amplifies the *S. aureus*-induced inflammatory response *in vitro*. **(A)** PPI network of 15 key genes. **(B)** The 4 most important hub genes were identified using the plugin MCODE of Cytoscape. **(C–F)** Expression levels of the four hub genes in keratinocytes after 3, 8, and 24 h of HKSA stimulation as measured by qRT-PCR. **(G–J)** qRT-PCR analysis of the four hub genes in keratinocytes transfected with miR939 or miRCtrl, followed by treatment with HKSA or PBS for 8 (h) **(K)** Immunofluorescence staining of MMP1 and MMP9 was performed on keratinocytes treated by miRCtrl, miR-939, miRCtrl&HKSA or miR-939&HKSA. The nucleus is stained with DAPI. 400x magnification, scale bars: 100 µm. **(L, M)** The mean fluorescence intensity of MMP1 and MMP9 of each field in different group is determined using ImageJ software in a blinded manner. The random 3 fields of each sample are scanned and then quantified. **(N–Q)** qRT-PCR analysis of the four hub genes in keratinocytes transfected with miR939 inhibitor or controls, followed by treatment with HKSA or PBS for 48 (h) The data are presented as mean ± SD. *P < 0.05, **P < 0.01, and ***P < 0.001 by One-way ANOVA test.

To determine the potential interactions among 15 key genes, we constructed a functional protein association network using STRING network analyses (**Figure 3A**). Moreover, the plugin MCODE identified the four most important hub genes, including three matrix metalloproteinases (MMP1, MMP3, and MMP9) and one cell surface adhesion receptor gene, ICAM1 (**Figure 3B**), which regulates leukocyte recruitment from the circulation (23). To validate the results obtained from the above bioinformatics analysis, we treated keratinocytes with HKSA at different times. The qRT-PCR results confirmed that the expression of MMP1, MMP3, MMP9, and ICAM1 was significantly upregulated by HKSA in a time-dependent manner (**Figures 3C–F**). Moreover, overexpression of the miR-939 significantly amplified the expression of MMP1, MMP3, MMP9, and ICAM1 following an 8-hour treatment of keratinocytes with HKSA (**Figures 3G–J**). To confirm these results, we further performed immunostaining of MMP1 and MMP9 after miR-939 transfection followed by HKSA stimulation. We also found that miR-939 significantly upregulate HKSA-induced MMP1 and MMP9 protein expression (**Figures 3K–M**). To determine whether the HKSA-induced endogenous miR-939 can act as positive feedback in keratinocytes, we transfected the keratinocytes with miR-939 blocking oligonucleotides that hybridize to mature miRNAs (inhibitors) followed by HKSA stimulation. Inhibition of miR-939 is not able to change the expression of MMPs at basal level. This may be due to the low expression level of miR-939 in psychological conditions. However, miR-939 inhibitor significantly decreased HKSA-induced MMP1, MMP3 and MMP9 expression, demonstrating that HKSA-induced miR-939 expression acts as positive feedback in keratinocytes (**Figures 3N–Q**). Together, these data suggest that miR-939 enhances the HKSA-induced inflammatory responses in human primary keratinocytes.

### miR-939 amplifies the S. aureus-induced atopic dermatitis phenotype *in vivo*

**Figure 4:**
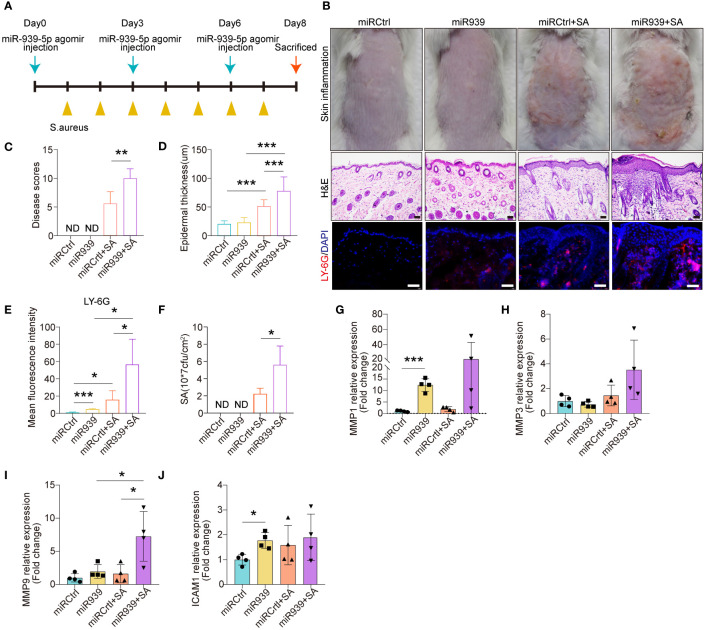
miR-939 amplifies *S. aureus*-induced atopic dermatitis phenotype *in vivo*. **(A)** Schematic of the animal study. **(B)** hsa-miR-939 agomir or corresponding negative control was mix with *in vivo*-jetPEI transfection reagent and intradermally injected to the mice back skin followed by colonization with *S. aureus* or treated with PBS. The above image is a representative photograph of a mouse skin lesion. The image in the middle is a corresponding H&E-stained section and below represents immunostaining for LY-6G. 200x magnification for H&E staining, 400x magnification for LY-6G immunostaining, scale bars: 100 µm. n = 4 mice per group. Skin disease scores **(C)**, epidermal thickness **(D)**, mean fluorescence intensity of LY-6G immunostaining **(E)** and the number of *S. aureus* colonized on the skin **(F)** were measured in each group. **(G–J)** qRT-PCR analysis of the expression levels of the four hub genes in each group. ND: not detected. The data are presented as mean ± SD. *P < 0.05, **P < 0.01, and ***P < 0.001 by One-way ANOVA test.

We further explored the physiological relevance of miR-939 in human keratinocytes and AD mice. We mixed either the miR-939 agomir or the control agomir with *in vivo*-jetPEI transfection reagent and administered it through intradermal injection into the mice’s skin followed by topical application of *S. aureus* (**Figure 4A**). Consistent with a previous study (9), *S. aureus* colonization was successfully established as an AD-like phenotype in mouse back skin (**Figures 4B, C**). The expression of AD-related cytokines, e.g. IL-13, TSLP and IL-6, were increased, while the antimicrobial peptide expression, e.g. mBD4, mBD14 and CAMP were deceased in *S. aureus* colonized skin (**Supplementary Figure S2**). Skin redness and epidermal thickness were significantly increased in the *S. aureus* colonization group (**Figures 4B, D**). Moreover, we also observed increased neutrophil infiltration in the skin dermis by *S. aureus* (**Figures 4B, E**). After injecting miR-939, *S. aureus* had a greater ability to colonize the skin of mice compared with the control mice (**Figure 4F**). This was evident by increased skin redness, epidermal thickness, and neutrophil infiltration in the miR-939+*S.aureus* group (**Figures 4B–F**). The increased *S. aureus* colonization and AD phenotype may be attributed, in part, to upregulated MMP1 and MMP9 expression in the skin (**Figures 4G–J**). Together, these data demonstrate that miR-939 amplifies the *S. aureus*-induced AD phenotype *in vivo*.

## Discussion

In this study, we established miRNA expression profiles in human primary keratinocytes after stimulation with heat-killed *S. aureus*. We focused on a highly upregulated miRNA by *S. aureus* stimulation, miR-939, and found that it was significantly upregulated in the epidermis of human AD lesions. We observed enhanced inflammatory responses in *S. aureus*-stimulated human primary keratinocytes. Overexpression of miR-939 increases the expression of matrix metalloproteinases to promote the colonization of *S. aureus* and exacerbate *S. aureus*-induced AD-like skin inflammation *in vitro* and *in vivo*. Moreover, inhibition of miR-939 decreased *S. aureus*-induced matrix metalloproteinases expression in keratinocytes. These results identified miR-939 as an important regulator in the immunopathogenesis of AD, which serves as a positive feedback loop after *S. aureus* stimulation and exacerbate matrix metalloproteinases expression.

MiR-939 exert diverse functions in different cells. Overexpression of miR-939 increased proliferation, migration, and invasion in glioma cell lines and increased miR-939–5p was associated with poor prognosis in glioma patients (16). The oncogenic role of miR-939 was confirmed in non−small cell lung cancer (NSCLC) indicating that miR−939 knockdown inhibits cell proliferation and invasion in NSCLC cell lines (17). MiR-939 also inhibited the expression of proinflammatory genes and decreased inflammation-induced apoptosis of endothelial cells (19). MiR-939 also exert specific roles in the regulation of innate immune response. McDonald et al. demonstrate that miR-939 inhibits the expression the proinflammatory cytokines in THP1 cells, e.g. IL-6, TNFa, NOS2 NOS2A and NFκB2 mRNAs (24). Hou et al. showed that MiR-939 abolished vascular integrity and repressed angiogenesis through directly targeting γ-catenin (25). In the present study, we demonstrated that miR-939 increased the expression of MMPs to enhance *S. aureus* colonization.

In contrast to coagulase-negative staphylococci (CoNS), such as *S. epidermidis* and *S. hominis*, *S. aureus* is characterized by a higher abundance of virulence factors. Our findings indicate that *S. aureus*, but not *S. epidermidis* or *S. hominis*, induces the expression of miR-939 which further enhances MMPs expression. Phenol-soluble modulins (PSMs), a family of amphipathic, α-helical peptides, serve as key virulence determinants, particularly in highly virulent *S. aureus* strains. PSMs, including PSMα, have been demonstrated to induce the expression of a wide range of pro-inflammatory chemokines and cytokines. Nakagawa et al. demonstrated that *S. aureus* produces PSMa, which induces keratinocyte damage and releases IL-1a and IL-36a, thereby triggering a downstream IL-17-dependent inflammatory response (26). The PSMs secreted by *S. aureus* may contribute to the observed synergistic effect in this study. Investigating the impact of PSMs on miR-939 and MMPs expression in human keratinocytes is a direction for future research.

Matrix metalloproteinases (MMPs) are a large family of calcium-dependent, zinc-containing endopeptidases, which are responsible for the degradation of the extracellular matrix (ECM) (27). A proteomics analysis of AD revealed that inflammatory markers, including matrix metalloproteinases (MMP1, MMP3, MMP9, and MMP12), Th2-type cytokines, and chemokines, are significantly upregulated in both lesion and non-lesion skin in patients with AD compared with healthy normal skin (28, 29). The upregulation of MMPs contribute to the breakdown of structural proteins in the skin, such as collagen and elastin, which are essential for maintaining the integrity of the skin barrier (30). Moreover, MMPs have been implicated in the modulation of sensory nerve function and itch sensation (31). Increased MMP activity in the skin may contribute to the neuroinflammatory processes underlying pruritus (itch) in AD. Disruption of the skin barrier allows allergens, irritants, and pathogens, such as *S. aureus* to penetrate the skin more easily, leading to inflammation and exacerbation of AD symptoms (32). In the present study, we demonstrated that miR-939 promotes *S. aureus*-induced MMP expression and colonization of *S. aureus* was increased in mice back skin after miR-939 administration. This phenomenon may be due to further damage of the skin barrier by MMPs.

MiRNAs normally bind to the 3’ untranslated region (UTR) of messenger RNA (mRNA) to induce degradation and/or translational repression (33). However, multiple miRNAs positively regulate gene expression by interacting with the promoter to trigger the recruitment of transcription factors and RNA-Polymerase-II. For example, miRNA-551b interacts with RNA Pol II to recruit the TWIST1 transcription factor to the STAT3 promoter to activate STAT3 transcription (34). MiR-373 interacts with the E-cadherin promoter to induce gene expression by recruiting RNA Pol II (35). In the present study, we observed a similar gene activation phenomenon with miR-939 in HKSA-stimulated keratinocytes. MiR-939 may bind to gene promoters; however, the precise mechanism through which miR-939 promotes MMP expression requires further study.

In conclusion, we demonstrated that *S. aureus* increases miR-939 expression *in vitro* and *in vivo*. MiR-939 enhances the expression of matrix metalloproteinase genes to promote the colonization of *S. aureus* and exacerbates *S. aureus*-induced AD-like skin inflammation. The results suggest that decreasing miR-939 levels in the skin may be a strategy to control the immunopathogenesis of AD driven by *S. aureus* colonization and infection. Thus, miR-939 may be a promising candidate for AD treatment.

## Methods

### Human samples

Three AD and three normal healthy samples were obtained from biobank of Institute of Dermatology, Chinese Academy of Medical Sciences, Jiangsu Biobank of Clinical Resources (BM2015004). All patients had signed informed consent for donating their samples. The study was approved by the Research Ethics Committee of the Hospital of Dermatology, Chinese Academy of Medical Sciences & Peking Union Medical College (2023-KY-033).

### Mouse model and *in vivo* studies

Seven-week-old male BALB/c mice were purchased from GemPharmatech (Nanjing, China) and acclimatized for one week. The mice were depilated on their backs and allowed to rest for one day to restore the skin barrier. The hsa-miR-939–5p agomir and corresponding negative control (GenePharma, Suzhou, China) were transfected into the mouse skin using *in vivo* jetPEI via intradermal injection, which is an *in vivo* transfection reagent (Polyplus), three times per week according to the manufacturer’s protocol. During this period, the mice were externally colonized with *S. aureus* by placing ten million CFU of *S. aureus* onto sterile gauze, which was then immobilized on the dorsal skin of the mice with a transparent bio-occlusive dressing (Tegaderm; 3M). On the 8th day, the mice were photographed and euthanized. The skin was collected for various analyses, including qRT-PCR, H&E staining, immunofluorescence, and *S. aureus* enumeration. A disease score was determined by the cumulative scores of erythema, scale, edema and lichenosis in skin inflammation, with each being designated as none (0), mild (1), moderate (2), or severe (3). The animal studies were conducted under approved protocols by the Animal Use and Care Committee of the Hospital of Dermatology, Chinese Academy of Medical Sciences & Peking Union Medical College (2023-DW-010).

### *S. aureus* culture and enumeration

*S. aureus* (USA 300 LAC) was a gift from Professor Yuping Lai (East China Normal University). It was cultured with TSB (Solarbio, Beijing, China) in a shaker at 37°C and 220 rpm. The mixed *S. aureus* bacterial solution was diluted in a 1:10 concentration gradient. Different concentrations of bacterial solution as well as TSB were added to 96-well enzyme-labeled plates at 200 µl per well and the absorbance was read at 600 nm using a microplate reader (BioTek, Synergy™ H1). In addition, 10 µl of different concentrations of the bacterial solution was inoculated into the TSA (Solarbio, Beijing, China) plate and incubated at 37°C for 24 h to count CFUs. Finally, a standard growth curve of *S. aureus* was plotted based on the OD600 values and CFU counts for the different concentrations of *S. aureus*. To measure the concentration of *S. aureus*, one CFU was placed in 10 ml of a TSB in a shaker at 37°C and 220 rpm for 24 h. The next day, 10 µl of TSB was collected and incubated in 5 ml of fresh TSB for 3–4 h. The absorbance was measured and the concentration of *S. aureus* was calculated from the standard growth curve. To determine the number of *S. aureus* in colonized skin, skin of the same size was collected, homogenized in cold PBS, diluted, and placed onto TSA plates. The number of CFUs was determined after incubating at 37°C for 24 h.

### qRT-PCR

Frozen mouse skin was homogenized with a Tissuelyser (JingXin, Shanghai, China). Total RNA was extracted from tissue homogenates and cells using RNAiso Plus (Takara). RNA quality and concentration were determined with a NanoDrop™ One/OneC (Thermo Fisher Scientific). Complementary DNA (cDNA) was synthesized using PrimeScript™ RT Master Mix (Takara) based on the manufacturer’s instructions. qRT-PCR was performed using TB Green® Premix Ex Taq™ II (Takara). For miRNA quantification, Bulge-loop miRNA qRT-PCR Primer Sets (one RT primer and a pair of qPCR primers for each set) specific for hsa-miR-939–5p were designed by RiboBio (Guangzhou, China). The expression of mRNA or miRNA was normalized to Gapdh or U6, respectively. The relative mRNA/miRNA expression was calculated using the 2^−ΔΔCt^ method.

### FISH

FISH was done using a miRNA FISH Kit with FISH probes (GenePharma, Suzhou, China). Briefly, paraffin sections were deparaffinized, digested with proteinase K, denatured at 73°C, hybridized overnight at 37°C, washed, and stained for nuclei. All images were collected at a magnification of 400X using a fluorescence confocal microscope (Olympus, BX53, Tokyo, Japan). DAPI (Invitrogen, Thermo Fisher Scientific) and streptavidin Cy3 channels were used for signal detection.

### Histology and immunofluorescence

Mouse skin tissues were fixed in formalin, embedded in paraffin, sectioned, and stained with hematoxylin and eosin. To calculate the average epidermal thickness of the mouse sections, we randomly selected five epidermal area on one image to measure the thickness by using ImageJ software. We totally analyzed four images from four mice. For immunofluorescence, fluorescence detection of neutrophil surface marker proteins in paraffin sections of mouse dorsal skin was done using an anti-Ly-6G/Ly-6C antibody (mouse monoclonal antibody; Thermo Fisher Scientific), followed by an anti-mouse AF488-conjugated secondary antibody (Invitrogen A32723). The fluorescent signal was observed using a fluorescence confocal microscope.

### NHEK culture, transfection, and stimulation

NHEKs (Cat: FC0025, Lot 09213, Lifeline Cell Technology) were cultured in DermaLife Basal Medium (Lifeline Cell Technology) supplemented with DermaLife K lifefactor kit and 1× antibiotics [penicillin (100 U/ml), streptomycin (100 U/ml); Thermo Fisher Scientific] at 37°C and 5% CO_2_. This donor is a 56-year-old Caucasian male. To study the effect of TLRs ligands on miR-939 expression, Flagellin (0.5ug/ml, InvivoGen), LPS (5ug/ml, InvivoGen), poly(I:C) (300ng/ml, InvivoGen), Zymosan (50ug/ml, InvivoGen) and Pam3CSK4 (10ug/ml, InvivoGen) was used to treat keratinocyte for 24 hours. The expression of miR-939 was measured by qRT-PCR. Before stimulating keratinocytes with HKSA (Thermo Fisher Scientific) or TLR ligands (Thermo Fisher Scientific), 20 nmol/L of hsa-miR‐939 mirVana miRNA mimic or mirVana miRNA mimic negative control #1 (Invitrogen, Thermo Fisher Scientific) was transfected into keratinocytes using Lipofectamine RNAiMAX (Thermo Fisher Scientific) when the confluence reached 50% to 60%. The final concentration of HKSA was 1 x 10^8^ CFU/ml for RNA-sequencing and *in vitro* studies, whereas the final concentration of various TLR ligands was based on the manufacturer’s instructions. NHEKs were only used for experiments between passages three and five.

### Bioinformatic analysis

To determine the relationship between the samples, we performed PCA using the R packages “factoextra” and “FactoMineR”, based on the normalized FPKM values for each sample. Raw Count values were used to perform a DEG analysis of the RNA-sequencing data using the R package “Deseq2”. Briefly. the first step was to construct a DESeqDataSet object using the function DESeqDataSetFromMatrix. Then, the function DESeq was used for the difference analysis. To acquire DEGs regulated by HKSA or miR-939, genes with parameters of |logFC| > 0.5 and P-value <0.05 were considered significantly changed. The package “ggplot2” was used to generate a volcano map. We used an online tool (https://maayanlab.cloud/Enrichr/) for KEGG and Elsevier enrichment analysis of the DEGs, and the top five enriched pathways are shown as bar graphs. The heatmap was generated with DEGs from inflammation-related signaling pathways upregulated by miR-939 using the “ComplexHeatmap” package. Protein–protein interactions were determined using STRING (https://cn.string-db.org/) and visualized by Cytoscape (version 3.9.0). Four hub genes were identified using the plugin MCODE for Cytoscape.

### RNA-sequencing

RNA-sequencing was done using HKSA-stimulated keratinocytes transfected with hsa-miR-939–5p mimic or the corresponding negative control. Briefly, total RNA was extracted and RNA quality was assessed. Then, mRNA was enriched using Oligo(dT), fragmented, and a cDNA Library was constructed using the NEB Next Ultra RNA Library Prep Kit for Illumina (NEB #7530, New England Biolabs, Ipswich, MA, USA). The resulting cDNA library was sequenced using an Illumina Novaseq6000 by Gene Denovo Biotechnology Co. (Guangzhou, China). The reads were subjected to quality control, sequence alignment, and gene abundance quantification by the FPKM and Count methods. The FPKM method eliminates the effects of varying gene lengths and sequencing data volume on gene expression calculations. Thus, the calculated gene expression may be used directly for subsequent bioinformatics analyses.

### Quantification and statistical analysis

Statistical analysis was performed with GraphPad Prism version 8.0 (GraphPad). All data were presented as means ± SEM. Statistical significance between groups was determined using either a two-tailed Student’s t-test or ANOVA analysis, by using GraphPad Prism 8 (GraphPad software Inc, California, USA). P values <0.05 were considered statistically significant.

## Data availability statement

The datasets presented in this study can be found in online repositories. The names of the repository/repositories and accession number(s) can be found below: (https://www.ncbi.nlm.nih.gov/geo/, GSE249837).

## Ethics statement

The studies involving humans were approved by the Research Ethics Committee of the Hospital of Dermatology, Chinese Academy of Medical Sciences & Peking Union Medical College. The studies were conducted in accordance with the local legislation and institutional requirements. The participants provided their written informed consent to participate in this study. The animal study was approved by the Animal Use and Care Committee of the Hospital of Dermatology, Chinese Academy of Medical Sciences & Peking Union Medical College. The study was conducted in accordance with the local legislation and institutional requirements.

## Author contributions

JW: Software, Visualization, Methodology, Formal analysis, Data curation, Writing – original draft. YH: Data curation, Formal analysis, Methodology, Validation, Visualization, Software, Writing – original draft. XW: Writing – review & editing, Visualization, Supervision, Resources, Methodology, Investigation, Formal analysis, Conceptualization. DL: Writing – review & editing, Writing – original draft, Supervision, Software, Methodology, Investigation, Funding acquisition, Conceptualization.
