# Supplementary material for: MicroRNA-939 amplifies Staphylococcus aureus-induced matrix metalloproteinase expression in atopic dermatitis
Source: Front Immunol. 2024 Jun 5;15:1354154. doi: 10.3389/fimmu.2024.1354154 (PMC11188349; doi:10.3389/fimmu.2024.1354154)
Supplement: Supplementary file 1 [file DataSheet_1.pdf]

Supplement Materials

Figure S1

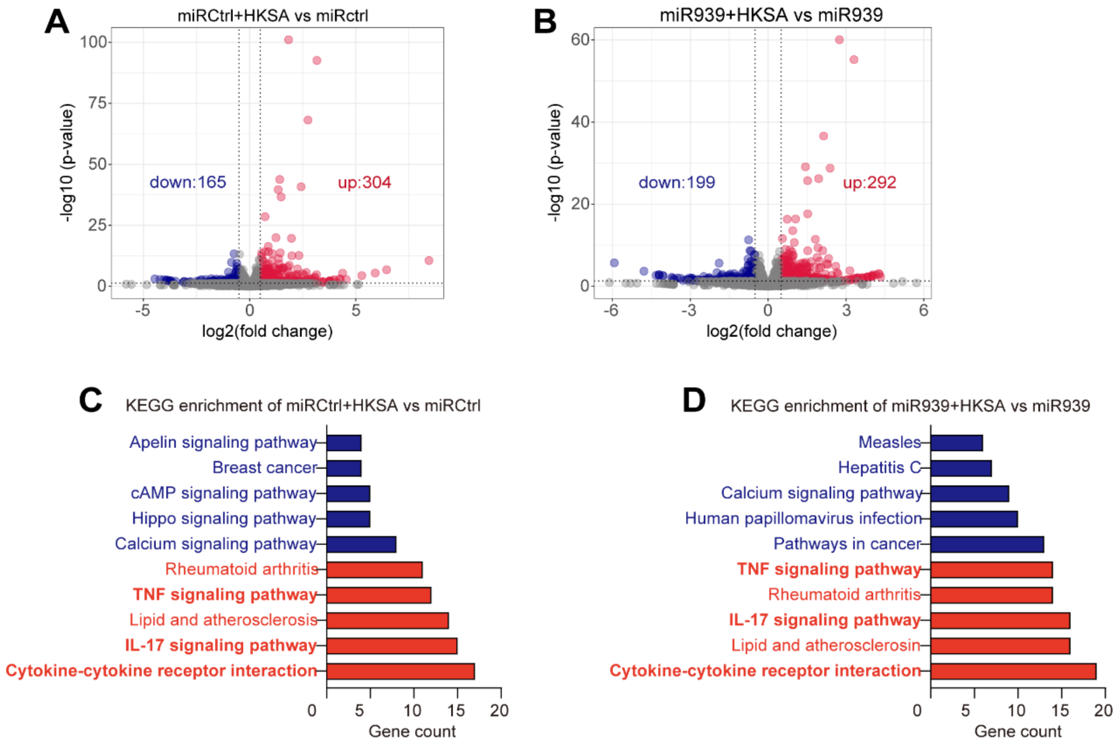

Figure S1. Transcriptome analysis shows that HKSA induces the inflammatory response of keratinocytes. Keratinocytes transfected with hsa-miR-939-5p mimic or the corresponding negative control, followed by treatment with HKSA or PBS for 8 h were subject to RNA sequencing. Differentially expressed genes in miRCtrl+HKSA compared with miRCtrl (A), and in miR939+HKSA compared with miR939 (B) are shown in volcano maps. Top KEGG pathways for the DEGs regulated by miRCtrl+HKSA (C) and miR939+HKSA (D) are shown in bar charts.

Figure S2.

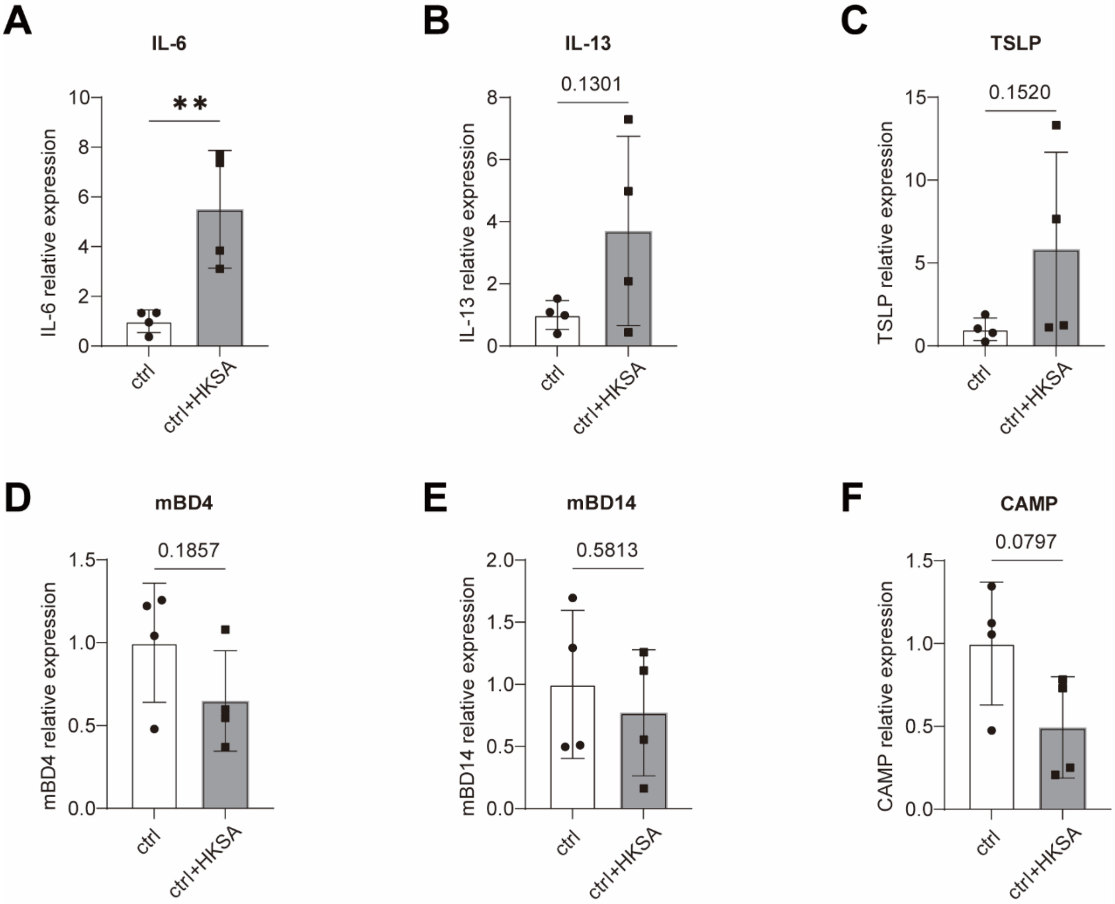

Figure S2. Dysregulated expression of AD-associated inflammatory cytokines and AMPs in mouse skin lesion areas induced by *S. aureus.* The mice were externally colonized with S. aureus or PBS for 8 days. On the 8th day, their skin was collected for qRT-PCR to detect expression of AD-associated inflammatory cytokines (IL-6, IL-13, TSLP) and AMPs (mBD4, mBD14, CAMP). *P < 0.05, **P < 0.01 by a Student's t-test.

Table S1. The primers used in this study.

Name	Species	Forward	Reverse
GAPDH	human	GGTGTGAACCATGAGAAGTATGA	GAGTCCTTCCACGATACCAAAG
MMP1	human	GCCTTCCAACTCTGGAGTAATG	GAGCTGCAACACGATGTAAGT
MMP3	human	GGAGATGCCCACTTTGATGA	AGGTCCATAGAGGGACTGAAT
MMP9	human	CTACCACCTCGAACTTTGACAG	GCCATTCACGTCGTCCTTAT
ICAM1	human	CCCGAGCTCAAGTGTCTAAAG	GCTGCTACCACAGTGATGAT
GAPDH	mouse	AATGGTGAAGGTCGGTGTG	GTGGAGTCATACTGGAACATGTAG
MMP1	mouse	GTGCCTGATGTGGGTGAATA	TGTCAGCAGTGCCATCATAG
MMP3	mouse	GAGGAAATCCCACATCACCTAC	GGGACAAAGTCTCCATGTTCTC
MMP9	mouse	GGACGTCAAATGTGGGTGTA	GCACACCAGAGAACTCCTTATC
ICAM1	mouse	TACGTGTGCCATGCCTTTAG	CTCCTGAGCCTTCTGTAACTTG
IL6	mouse	GATGGATGCTACCAAACTGGA	CCTTCTGTGACTCCAGCTTATC
IL13	mouse	GTGCCAAGATCTGTGTCTCTC	TATCCTCTGGGTCCTGTAGATG
TSLP	mouse	AACATCACACAAGACCAGACTC	GGTTACAGAGGCCATGCAATA
mBD4	mouse	AAGCAGCAGGTGAAGCAAATC	TTCTACTTGTGTCCGTGGGA
mBD14	mouse	CTCATCTTGTTCTTGGTGCCT	CGACCGCTATTAGAACATCGAC
Camp	mouse	GTCTGTGAGGTTCCGAGTGA	AGGCTCGTTACAGCTGATGTC
